# Antiangiogenic Effects of VH02, a Novel Urea Derivative: In Vitro and in Vivo Studies

**DOI:** 10.3390/molecules21091258

**Published:** 2016-09-21

**Authors:** Suwadee Phowichit, Miho Kobayashi, Yuriko Fujinoya, Yasufumi Sato, Kingkarn Sanphanya, Opa Vajragupta, Linda Chularojmontri, Suvara K. Wattanapitayakul

**Affiliations:** 1 Department of Pharmacology, Faculty of Medicine, Srinakharinwirot University, 114 Sukhumvit 23, Bangkok 10110, Thailand; swdbell@hotmail.com; 2 Department of Vascular Biology, Institute of Development, Aging and Cancer, Tohoku University, 4-1 Seiryo-machi, Aoba-ku, Sendai 980-8575, Japan; miho.kobayashi.c1@tohoku.ac.jp (M.K.); fujinoya@idac.tohoku.ac.jp (Y.F.); yasufumi.sato.b3@tohoku.ac.jp (Y.S.); 3 Department of Pharmaceutical Chemistry, Faculty of Pharmacy, Mahidol University, 447 Sri Ayudhya Road, Bangkok 10400, Thailand; kingkansan@gmail.com (K.S.); opa.vaj@mahidol.ac.th (O.V.); 4 Department of Preclinical Sciences, Faculty of Medicine, Thammasat University, 95 Paholyotin Rd, Klongluang, Pathumthani 12120, Thailand; ning.phd@gmail.com

**Keywords:** vascular endothelial growth factor receptor 2 (VEGFR2), structure-based drug design, urea derivatives, angiogenesis, endothelial cells

## Abstract

Vascular endothelial growth factor receptor 2 (VEGFR2) is a vital target for therapeutic intervention in cancer. We have recently described a computer-based drug design for a small molecule VEGFR2 inhibitor named VH02 (1-((1-(1*H*-indazol-6-yl)-1*H*-1,2,3-triazol-4-yl)methyl)-3-(3-chloromethylphenyl)urea). This study aimed to further explore the anti-angiogenic activity of VH02 both in vitro and in vivo. The in vitro assays include cell viability, capillary-like tube formation, MMP activity, and western blot analyses of signaling through VEGFR2 while the in vivo anti-angiogenic response were performed to evaluate the effect on vascularization in Matrigel plug applied in C57BL/6L mice. VH02 reduced angiogenesis behavior of EA.hy926 including cell viability, migration, adhesion, capillary-like tube formation, and *MMP-2* activity induced by VEGF. Furthermore, VH02 regulated angiogenesis by directly inhibiting VEGFR2 on Tyr1175 signaling pathway leading to the inhibition of Akt-mediated cell survival and migration. Disruption of phosphorylation at VEGFR2-Tyr1175 by VH02 abolished FAK-Tyr397 signaling but not phosphorylation of p38 MAPK. This suggests that blockade of FAK by VH02 apparently associated with reduction of endothelial cell motility. Actin cytoskeleton rearrangement was diminished by VH02 in human endothelial cells. The anti-angiogenic effect of VH02 was confirmed in the in vivo model, revealing the reduction of vascular density in Matrigel plug after VH02 treatment. Additionally, the pericyte-like cells surrounding blood vessels in the plugs were significantly reduced as well as vascular density and p-Akt intensity. Our findings indicate that VH02 successfully inhibits VEGF-induced angiogenesis both in vitro and in vivo models. The compound could be further developed as an antiangiogenesis agent for cancer therapy.

## 1. Introduction

Angiogenesis is an important facilitator for the development of solid cancer by sprouting new blood vessels from pre-existing endothelium to transport growth factors, oxygen, and various nutrients to tumors. The process of angiogenesis is complex requiring many inducers and receptors; among those, the vascular endothelial growth factor (VEGF) family members are key signal mediators that control endothelial cells in many aspects, particularly, in supporting tumor invasion and metastasis [1]. The VEGF subtype A (VEGFA) supersedes other family members (VEGFB, VEGFC, VEGFD) in the field of antiangiogenesis therapy because the first generation of drugs available in the market was targeting VEGF (commonly referred to VEGFA) and VEGF receptors (VEGFR).

VEGF specifically binds to VEGFR1 (Flt-1) or VEGFR2 (Flk-1/KDR), presented on the cell surface of endothelial cells. However, VEGFR2 is the main signal transducer responsible for angiogenesis because it has a strong intrinsic kinase activity and plays a decisive role in the regulation of tumor angiogenesis [2]. The interaction of VEGF with VEGFR2 causes cell proliferation, survival, migration, capillary-tube formation and permeability increase that occurs in rapidly growing tumors [3]. VEGF/VEGFR2 signaling pathway is initiated by the dimerization of VEGFR2 and subsequent phosphorylations of several tyrosine residues, e.g., Y951, Y1175, and Y1214. Among these residues, VEGFR2-Tyr1175 is the major autophosphorylation site following VEGF binding that controls angiogenic responses in vascular endothelial cells [4,5]. Phosphorylation of VEGFR2-Tyr1175 regulates downstream signaling through phospho-Akt at Ser473 promoting endothelial cell survival and migration [6,7]. The Akt activation is an almost indispensible signal transduction for regulating endothelial cell survival and migration through the PI3K/AKT and eNOS pathways [4,8]. The endothelial cell motility activated by VEGFR2 also associates with focal adhesion molecule (FAK) via the interaction of VEGFR2 and Shb adaptor protein. Particularly, VEGF-induced FAK phosphorylation at Tyr 397 in endothelial cells is a highly conserved autophosphorylation site by transmitting different signals to their targets. It can sustain an increased formation of actin stress fibers and new focal adhesion resulting in several biological processes, including endothelial cell adhesion, migration, invasion, tube formation and survival [6,9,10,11].

The first antiangiogenesis drug bevacizumab (a monoclonal antibody for VEGFA) was launched in 2004 and many small molecules that inhibit the activity and signaling cascade of VEGF receptors (VEGFRs) have been developed and approved by the US FDA for the treatment of various types of cancer [12]. Currently, the antiangiogenesis drugs against VEGF are mostly effective in clinical practice when combined in chemotherapy [13,14,15,16]. The small molecule VEGFR2 tyrosine kinase inhibitors (TKIs, e.g., sorafenib, sunitinib, pazofinib) usually have urea derivatives in their chemical structures which are related to their biological effects [17]. Sorafenib, an orally available angiogenesis drug for multi-tyrosine kinase inhibition (VEGFR2, VEGFR3, PDGFR-beta and c-kit), is a representative compound that comprises a biaryl urea in its structure [18]. It was approved by US FDA for advanced renal cancer in 2005. Thus, the development of novel VEGFR2 inhibitors using structure-based drug design targeting high affinity for VEGFR2 is of interest. We have recently reported synthesis of a small molecule VEGFR2 tyrosine kinase inhibitor VH02 or 1-((1-(1*H*-indazol-6-yl)-1*H*-1,2,3-triazol-4-yl)methyl)-3-(3-chloromethylphenyl)urea by using a back to front approach from in silico drug design [19]. VH02 is a type II kinase inhibitor which is more selective to VEGFR2 than type I kinase inhibitors. The core motif of VH02 contains 1-((3-chloromethyl) phenyl)-3-(2-propynyl)urea that is buried in the back pocket of VEGFR2. The design of VH02 allows for strong hydrogen bonding to the kinase domain of VEGFR2 while two bonds interact with the key residues at the ATP binding site (front pocket) namely Glu917 and Cys919. Thus, we aim to confirm and evaluate the antiangiogenic activity of VH02 both in vitro and in vivo by targeting the key signals of VEGF-induced angiogenesis. We explored VEGFR2-Tyr1175 signaling pathway on the activation of Akt, FAK, and p38 in human endothelial cells EA.hy926. The in vivo mouse model of Matrigel plug assay was used to evaluate the effect of VH02 on vascular density and α-SMA coverage of the vessels. In this study, we found that VH02 inhibited angiogenesis upon VEGF stimulation both in vitro and in vivo which has high potential for further lead optimization as a cancer therapy agent.

## 2. Results

### 2.1. VH02 Inhibited VEGF-Induced EA.hy926 Cell Viability

**Figure 1:**
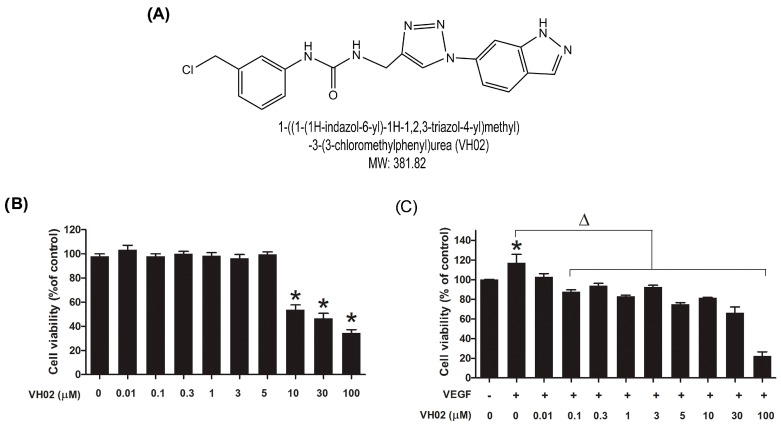
Effects of VH02 on EA.hy 926 cell viability. EA.hy 926 cells were treated with various concentrations of VH02 and co-incubated with or without VEGF (25 ng/mL) for 24 h. Cell viability was measured by MTT assay as described in the Materials and Methods section. (**A**) Chemical structure of VH02; (**B**) Cytotoxic effect of VH02 on the endothelial cells in the absence of VEGF (25 ng/mL); (**C**) Cytotoxic effect of VH02 in the endothelial cells in the presence of VEGF (25 ng/mL). * *p* < 0.05 compared with control (vehicle treated cells); Δ *p* < 0.05 compared with VEGF treated cells (VEGF control).

VH02, a small molecule VEGFR2 TKI (Figure 1A) had a negative influence on endothelial cell viability under serum starvation and VEGF stimulation. Under low serum condition, VH02 significantly caused endothelial cell death at the concentrations 10 μM or higher (Figure 1B). While VEGF (25 ng/mL) alone significantly increased cell viability to 117.1% ± 8.7% the co-incubation with VH02 significantly inhibited VEGF-induced endothelial cell viability at all concentrations tested (Figure 1C). Thus, further experiments were performed using the maximal concentration of VH02 at 5 μM.

### 2.2. VH02 Inhibits VEGF-Stimulated Cell Migration, Adhesion and Capillary-Like Tube Formation

**Figure 2:**
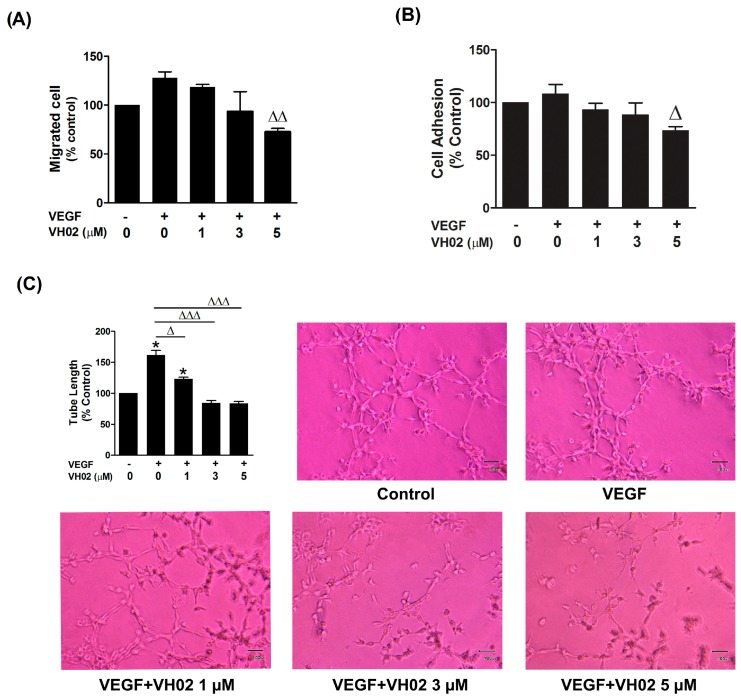
Influence of VH02 on VEGF-induced endothelial cell migration, cell adhesion and tube formation. (**A**) VH02 inhibited VEGF-induced EA.hy 926 migration. EA.hy 926 were allowed to migrate from the upper chamber of transwells plate to the lower chamber treated with or without VEGF (25 ng/mL) and varying doses of VH02. After culturing for 16 h, the migrated cells were moved through the porous membrane and quantified by measuring intracellular esterase enzyme activity; (**B**) Adhesion of EA.hy 926 on Matrigel-coated plates. Effects of VH02 on cell adherence were measure by MTT assay at 2 h in the presence or absence of VEGF (25 ng/mL); (**C**) Effects of VH02 on VEGF-induced capillary-like tube formation. EA.hy 926 suspended in different concentrations of VH02-containing media were seeded onto a growth factor-reduced Matrigel. VEGF (25 ng/mL) was added to stimulate capillary-like tube formation. Representative images were captured at a magnification of 100X on an Olympus inverted microscope. Quantification of percentage of tube lengths was assessed at least six randomly selected fields by Cell B imaging software. Values were presented as mean ± SEM of at least three separate experiments. * *p* < 0.05 when compared to vehicle-treated cells (control); Δ *p* < 0.05; ΔΔ *p* < 0.01; ΔΔΔ *p* < 0.001 compared with VEGF-treated cells alone (VEGF control).

The effect of VH02 on cell migration was quantified by tracking the intracellular esterase enzyme activity of cells that passed through the porous membrane of a transwell chamber after 16 h. VH02 significantly inhibited VEGF-stimulated endothelial cell migration at the concentration 3 and 5 μM compared with VEGF group (Figure 2A). Adhesion of endothelial cells also associates angiogenesis and metastasis at an early attachment step. Under VEGF stimulation, VH02 decreased EA.hy 926 adhesion by approximately 35% when compared with VEGF alone (Figure 2B).

Capillary-like tubules are formed in later stage of angiogenesis which generated from complex multiple step process including proliferation, migration, adhesion, and differentiation of endothelial cells. The tubular network was closely aligned to form a branched polygonal cell network similar to a mesh-like structure in the presence of VEGF (Figure 2C). In contrast, when VH02 was added with VEGF, a network of capillary-like tube formation was progressively lost and the tubes were generated irregularly without lumen. Endothelial capillary tube formation was remarkably abrogated by VH02 in a concentration-dependent manner at as low dose as 1 μM. With VEGF stimulation, VH02 at the concentrations of 1, 3 and 5 μM significantly decreased tube lengths by 38.71%, 77.28%, and 78.02%, respectively (Figure 2C). 

### 2.3. VH02 Suppressed VEGF-Induced Gelatinase Activity

**Figure 3:**
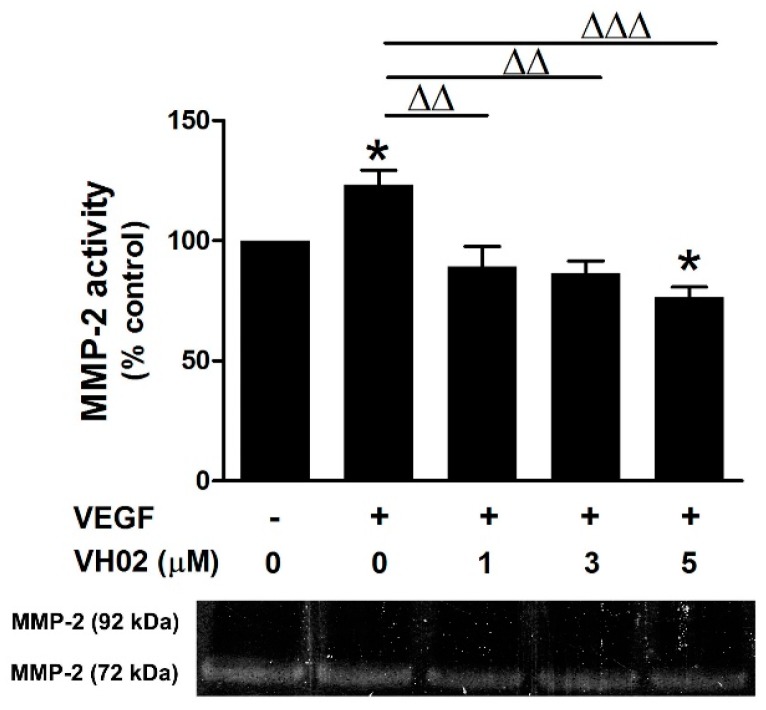
MMP-2 inhibitory effect of VH02 on EA.hy 926 cells. Representative gelatin zymography shows a clear visible band of MMP-2 activity at 72 kDa while MMP-9 activity at 92 kDa was not observed. The activity of MMP-2 was quantified by the band density and analyzed by Image J software. The data are expressed as percentage of reference control. * *p* < 0.05 versus vehicle control; ΔΔ *p* < 0.01; ΔΔΔ *p* < 0.01 versus VEGF (25 ng/mL) alone.

In the presence of VEGF, VH02 at various concentrations was able to suppress the gelatinase activity of MMP-2 in a dose-dependent manner, with strong significant inhibition at 1–5 μM (Figure 3). VH02 treatment gave a clearly visible band at 72 kDa but the band of MMP-9 (92 kDa) did not appear on the gelatin substrate.

### 2.4. VH02 Inhibited VEGFR2-Dependent Cell Signaling

**Figure 4:**
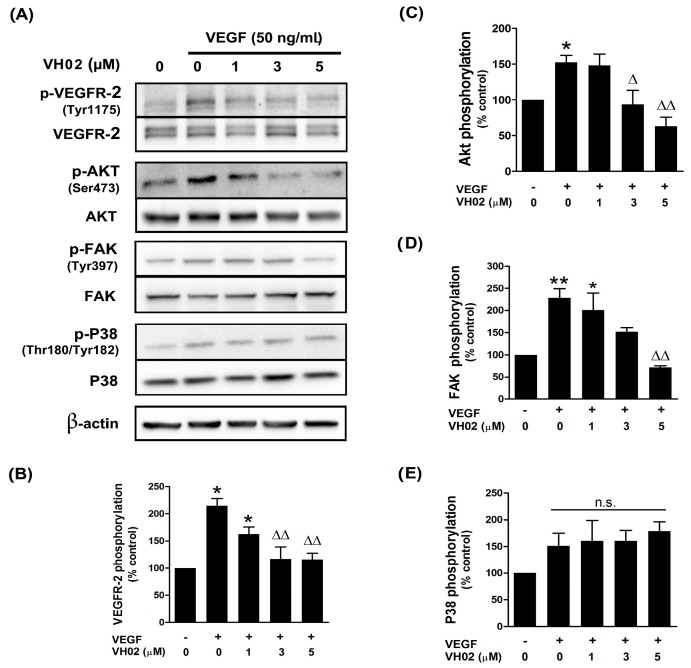
Effects of VH02 on VEGF-mediated signaling pathway through VEGFR2, Akt, FAK and P38 in EA.hy926 cells. The protein phosphorylation of VEGF receptor-2 (p-VEGFR2), Akt (p-Akt), FAK (p-FAK), and P38 (p-P38) was analyzed by western blot analysis as described in the Materials and Methods section. (**A**) Representative blots show protein phosphorylation signals relative to the non-phosphorylated proteins; (**B**) Changes in VEGFR2 phosphorylation; (**C**) Changes in Akt phosphorylation; (**D**) Changes in FAK phosphorylation; (**E**) Changes in P38 phosphorylation. Values were represented as mean ± SEM. * *p* < 0.05; ** *p* < 0.01 versus vehicle control; Δ *p* < 0.05; ΔΔ *p* < 0.01; n.s. non-significant versus VEGF alone.

VEGF treatment dramatically stimulated VEGFR2 autophosphorylation on Tyr1175 and downstream signaling by Akt and FAK (Figure 4).

The phosphorylation of VEGFR2 was decreased by VH02 at concentration 3, and 5 μM in a dose dependent manner, compared to VEGF alone (Figure 4A,B). The phosphorylation of AKT at Ser473 site, a key molecular target downstream of VEGFR2, was completely blocked by VH02 at both 3 and 5 μM under VEGF stimulation (Figure 4A,C). These results indicated that VH02 had an ability to abolish VEGF/VEGFR2/Akt-dependent endothelial cell survival. Next, we further identified the FAK activation on Tyr397, which is major target of endothelial cell motility and adhesion. VH02 at a concentration of 5 μM significantly inhibited FAK phosphorylation in presence of VEGF induction; however, there was no change in the phosphorylation of p38 MAPK (Figure 4A,D,E).

### 2.5. VH02 Influenced Actin Cytoskeleton of EA.hy 926

**Figure 5:**
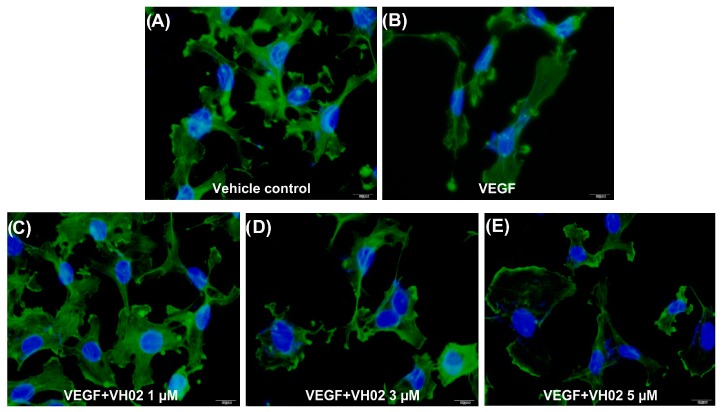
Immunofluorescence images of actin cytoskeleton in EA.hy926. Cells were treated with VH02 for 30 min, and then stimulated with or without VEGF (50 ng/mL) for 15 min. Actin fibers were visualized in cells fixed with 4% formaldehyde and tagged by anti-rabbit conjugated Alexa Fluor (green) as described in the Materials and Methods section. The nuclei were counterstained with DAPI (blue). All images were taken in the same exposure by Olympus immunofluorescence microscopy with 600× magnification. Scale bar = 10 μm. (**A**) Control cells under basal condition; (**B**) cells treated with VEGF (50 ng/mL); (**C**) cells treated with VH02 (1 μM) and VEGF (50 ng/mL); (**D**) cells treated with VH02 (3 μM) and VEGF (50 ng/mL); (**E**) cells treated with VH02 (5 μM) and VEGF (50 ng/mL).

The structures of vehicle-treated control cells were rearranged into membrane ruffles (Figure 5A). In response to VEGF stimulation, cells showed reorganization of actin characterized by prominent extension of stress fibers into parallel dispersion that evoked a migratory response at 15 min (Figure 5B). In contrast, pretreatment of cells with varying concentrations of VH02 for 30 min before adding VEGF inhibited stress fiber formation in a dose-dependent manner (Figure 5C–E). The actin cytoskeleton was changed in size and shape and a loss of polymerized actin was observed after treatment with VH02. These results suggested that VH02 could restrain rapid actin cytoskeleton rearrangement in VEGF-induced stress fiber formation and result in the inhibition of cell migration of EA.hy 926.

### 2.6. Confirmation of VH02 Action on HUVECs

**Figure 6:**
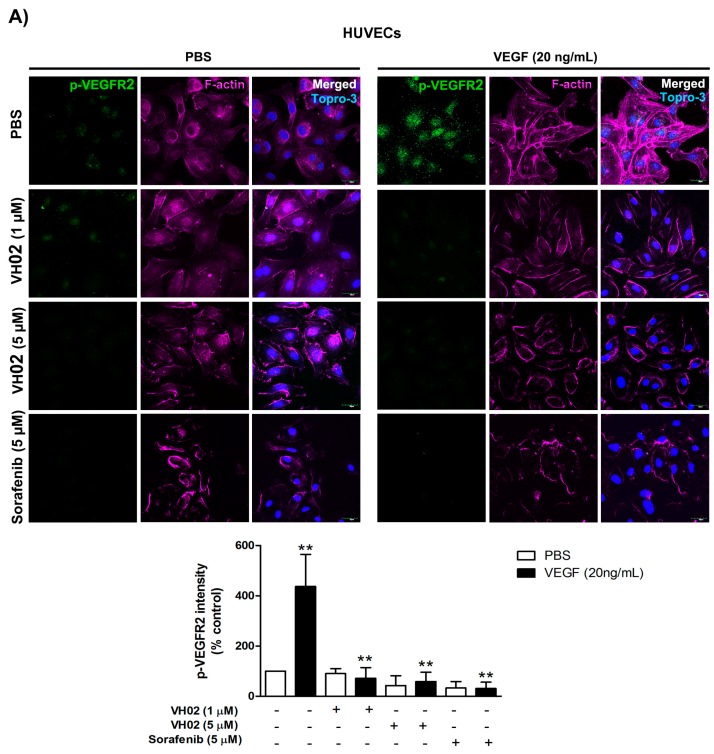

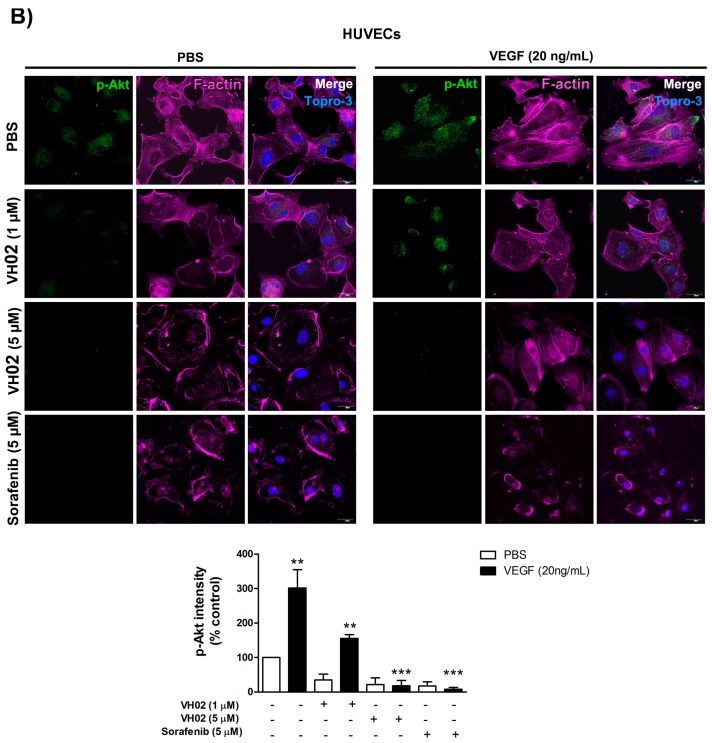
Effects of VH02 on VEGFR2 and Akt phosphorylation, and actin polymerization in HUVECs in normal control group (PBS) and VEGF-induced condition. (**A**) Representative confocal immunofluorescence images of HUVECs stained by fluorescent-tagged anti-p-VEGFR2 Tyr1175 antibody (green) in the presence and absence of VEGF treatment (control); (**B**) Representative confocal immunofluorescence images of HUVECs stained with fluorescent-tagged anti-p-Akt Ser473 antibody (green) in the presence and absence of VEGF treatment (control). HUVECs were pretreated for 30 min with VH02 or sorafenib as indicated in the figure. The slides were also counter-stained with antibodies against F-actin (pink). Cell nuclei were identified by Topro-3 (blue). All images were taken at the same exposure time by confocal microscopy with Z-stack projections (600× magnification). Scale bars, 30 μm. Fluorescence intensity values were represented as mean ± SEM of duplicate separate experiments with five randomly images from each group. ** *p* < 0.01, *** *p* < 0.001 compared with PBS of each pair of treatment (analyzed by two-way ANOVA).

The in vitro antiangiogenesis activity of VH02 was confirmed in HUVECs under serum starvation. VH02 (1 and 5 μM) effectively inhibited the phosphorylation of VEGFR2 and Akt under VEGF stimulation in a dose-dependent manner compared with VEGF alone. Sorafenib (5 μM) was performed as a reference drug for suppressing VEGF-induced phosphorylation of VEGFR2 and Akt. The levels of VEGF-stimulated VEGFR2 phosphorylation was significantly decreased from 437.09% ± 52.01% down to 71.88% ± 16.48%, 58.91% ± 15.61% and 31.11% ± 9.98% by VH02 (1 μM), VH02 (5 μM), and sorafenib (5 μM), respectively (Figure 6A). The intensity of p-VEGFR2 was influenced by both factor 1, VEGF stimulation, (PBS or VEGF) and factor 2, drug treatment, (vehicle, VH02, or sorafenib) which had a significant interaction at F(3,8) = 5.10, *p* < 0.05. The treatment with VH02 or sorafenib also inhibited VEGF-stimulated Akt phosphorylation from the level of 319.19% ± 42.79% down to 138.51% ± 23.18%, 17.59% ± 6.27%, and 12.84% ± 6.16%, respectively (Figure 6B). The changes in Akt phosphorylation were the results of the interaction between factor 1 (VEGF-primed condition) and factor 2 (drug treatment) which were observed at F(3,8) = 10.41, *p* < 0.01.

F-actin organization was visualized by staining with Alexa Fluor-conjugated phalloidin (Figure 6). In untreated HUVECs, F-actin staining was seen as a cortical network. VEGF promoted reorganization of the actin cytoskeleton into stress fibers along the longitudinal axis within 30 min. Co-treatment of HUVECs with 5 μM sorafenib with or without VEGF inhibited F-actin rearrangement. The F-actin cytoskeleton was changed in size and shape and the loss of F-actin polymerization was observed after cells were treated with 1 and 5 μM VH02. 

### 2.7. VH02 Blocked New Blood Vessel Formation in Matrigel Plug in Vivo

**Figure 7:**
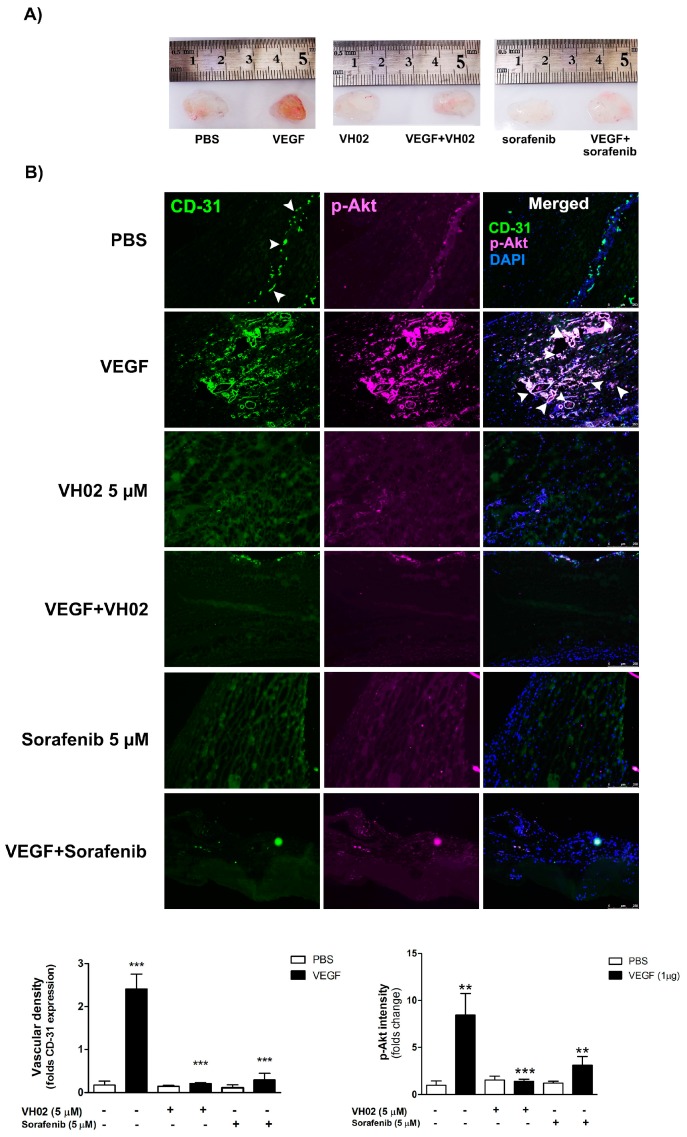

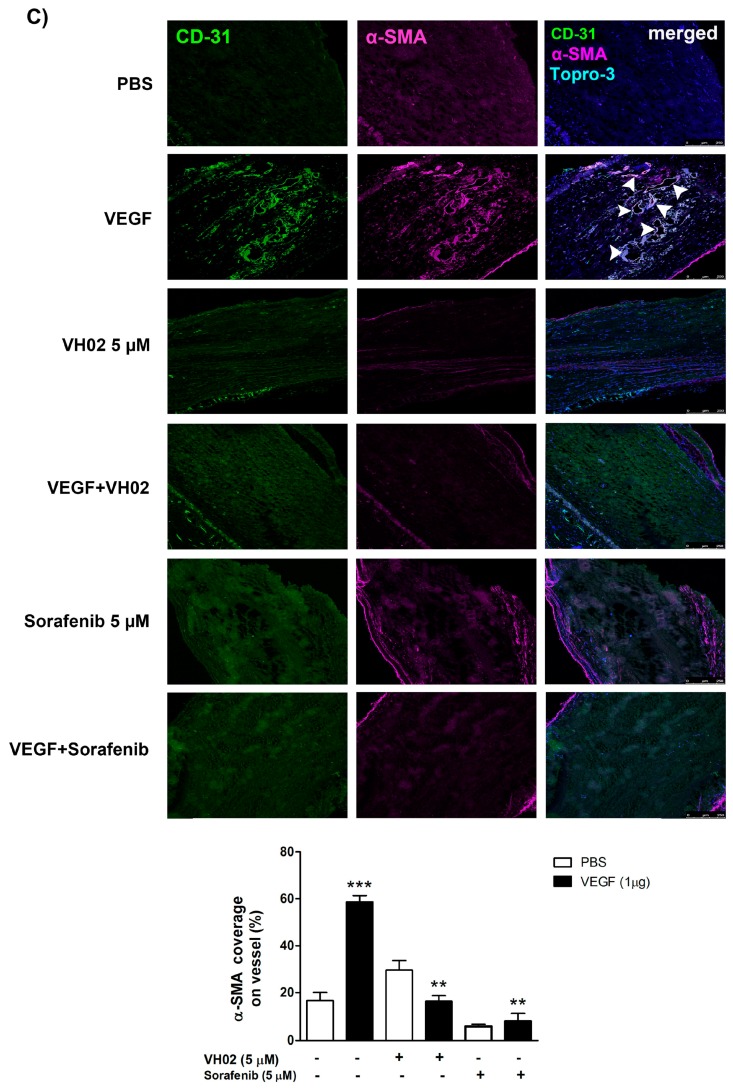
Antiangiogenesis effects of VH02 Matrigel plugs in a mouse model. Matrigel plugs were implanted into the hide limbs of mice with various treatments, including vehicle (PBS), VEGF (1 μg), VH02 (5 μM), sorafenib (5 μM), or combination as indicated in the figure. (**A**) Representative photos of Matrigel plugs from mice with different treatments; (**B**) Representative immunostaining of transverse cryosections of Matrigel plugs assessed with antibodies against CD-31 (green), p-Akt (pink), and DAPI (blue); (**C**) Representative immunofluorescence images of Matrigel plugs stained with antibodies against CD-31 and alpha-SMA. The cell nuclei were identified by Topro-3 iodide (blue) staining. Arrows indicate microvessel-like formation. Scale bars, 250 μm. The quantification of immunostaining intensity was performed in comparison with vehicle-treated groups (PBS). Values are mean ± SEM of at least six randomly selected images from each plug. ** *p* < 0.01, *** *p* < 0.001 compared with PBS of each pair of treatment (analyzed by two-way ANOVA).

The in vivo antiangiogenic activity of VH02 was shown in a Matrigel plug mouse model. After day 10, Matrigel plugs in the control group showed very sparse blood vessel formation. In contrast, plugs supplemented with 1 μg VEGF demonstrated dramatically increased new blood vessel formation marked by the dark red color in the plug (Figure 7A). Under VEGF stimulation, VH02 (5 μM) strongly reduced Matrigel plugs neovascularization by 12-fold, similar to the activity of sorafenib (5 μM). The Matrigel plugs showed enhanced immunofluorescent stained for CD31 after VEGF stimulation and were significantly blocked by VH02 or sorafenib (Figure 7B). There was a significant interaction effect between VEGF stimulation (factor 1) and treatments with VH02 or sorafenib (factor 2) on angiogenesis as detected by CD31 (F(2,12) = 28.12, *p* < 0.0001). Similarly, Akt phosphorylation was significantly reduced by VH02 treatment in the presence of VEGF. We also identified the pericyte-like cells within the plugs which were positively stained with anti-αSMA antibodies.

The pericyte-like cells were localized surrounding the endothelial cell layer. The coverage of blood vessels with pericyte α-SMA was significantly decreased after treatment with VH02 when compared to the VEGF group (Figure 7C). The interaction between factor 1 and factor 2 observed in the evaluation of p-Akt and αSMA were similar and found to be significant at F(2,12) = 7.31, *p* < 0.001.

## 3. Discussion

Antiangiogenic therapy has added value to traditional chemotherapy for solid tumors due to the suppression of tumor invasion and metastasis. Angiogenesis occurs when there is an imbalance of pro-angiogenic molecules secreted by the tumor mass, including VEGF165 isoform, which is one of most potent proangiogenic factors that sends signals through a surface receptor tyrosine kinase (VEGFR2) on endothelial cells. Binding of VEGF to VEGFR2 is a major mediator causing cascades of signals to initiate new blood vessel formation. Phosphorylation of VEGFR2 in each tyrosine site associates with key events including proliferation, migration, survival and permeation that are necessary for tumor angiogenesis. Suppressing VEGFR2 signaling through the inhibition of receptor tyrosine kinase phosphorylation is now an important target of antiangiogenic therapeutic strategies [13]. In this study, we explored the antiangiogenic activity of a novel VEGFR2 inhibitor, VH02, both in vitro and in vivo. We found that VH02 inhibited multiple steps of angiogenesis feature including endothelial cell viability, adhesion, migration, and tube formation. The main signaling cascade through VEGFR2 including phosphorylation of VEGFR2 and Akt was also inhibited both in vitro and in vivo. The vascular formation in Matrigel plugs implanted in mice was significantly inhibited along with the abrogation of α-SMA expression. This suggests that the novel antiangiogenic agent VH02 can potentially be a lead compound for further optimization in line of cancer therapeutic agents.

The type II kinase inhibitor VH02 was designed with the core structure of 1-(substituted)-3-prop-2-ynylureas with triazole linker using a back to front approach [19]. VH02 interacts with the side chains of a hydrophobic part of the back pocket including Ile888, Ile892, Val898, Val899, Leu1019, His1026, Ile1044, Cys1045 and Phe1047. The design of high affinity VEGFR2 inhibitors also extended to various structures including phthalazine derivatives [20], sulfonyl urea derivatives [21], guanidinium [22] and others which have similar side chains interactions. The in vitro cell culture studies revealed that VH02 differentially decreased endothelial cell viability; under VEGF stimulation, the endothelial cells were 100-fold more sensitive to VH02-induced cytotoxicity when compared to vehicle-treated cells. Thus, at low concentrations, this compound selectively inhibited endothelial cell growth in the environment of angiogenic drivers that favors the cytotoxic profile in the normal cell environment where VEGF levels are low. It also appears that VH02 dose-dependently inhibited early steps of angiogenesis, including endothelial cell migration and adhesion.

The endothelial migration processes during angiogenesis involve different stimulators such as chemotaxis (induced by chemoattractants), haptotaxis (activated by immobilized ligands), and mechanotaxis (driven by mechanical force, e.g., sheer stress) [11]. VEGF is an essential chemotactic factor during angiogenesis that initiates cell migration, cell adhesion, and the interaction of endothelial cells to ECM followed by the tubular network formation. The interaction of cell-matrix during adhesion and the tubular network formation on Matrigel mimicked the in vivo vascular formation. Early steps of endothelial cell migration depend on type IV collagenase activity of MMP-2 and MMP-9 [23]. These MMPs are major ECM proteolytic enzymes that degrade the basement membrane to facilitate cell invasion, sprouting and migration into the perivascular space. We demonstrated that VH02 effectively inhibited MMP-2 gelatinase activity in a dose-dependent manner, which was strongly associated with lower migratory activity due to the release of pro-angiogenic molecules that induced extracellular matrix degradation [24]. However, there was no change in MMP-9 activity which also plays an important role in metastasis and angiogenesis regulation. Our result was in agreement with other studies because MMP-9 is not inducible in starved conditions of EA.hy926 [25,26]. The inhibitory effect of VH02 was concordant with essential phenomena occurring during angiogenesis, which ultimately resulted in the inhibition of vascular tube formation. 

VH02 acts as a small molecule VEGFR2 tyrosine kinase inhibitor; thus, the downstream VEGFR2 signaling pathway was confirmed both in an endothelial cell line and HUVECs. The induction of VEGF/VEGFR2 signaling provides specific binding sites for several signaling protein such as PLC-γ (phospholipase C gamma), Shb (SH2 domain-containing adapter protein B, PI3K (phosphoinositide-3 kinase), Sck (Shc-like protein), or Grb2 (growth factor receptor-bound protein 2). These proteins require specific molecules including Akt, FAK, or P38MAPK, which are all involved in endothelial migration [6]. Phosphorylation of VEGFR2 Tyr1175 and downstream signaling to Akt and FAK is a major site for regulation of directional endothelial cell migration. We revealed that endothelial cells exposed to VH02 prior to VEGF stimulation inhibited the phosphorylation of VEGFR2 at Tyr1175 confirming the interference of VH02 in the process of VEGF signaling. In addition, VEGF directly mediated both endothelial cell survival and migration through Akt activation on Ser473 [7]. VH02 apparently decreased p-Akt Ser473, which leads to the inhibition of endothelial cell survival. VEGFR2-1175 activation also induced the FAK phosphorylation on Tyr 397 that is a crucial for control of F-actin fibers associated with cell migration [6,10,11]. As reported previously, downregulation of p-FAK is involved with angiogenic responses by suppressing adhesion molecules and migration via dynamic cytoskeleton organization [27,28]. Actin rearrangement plays a crucial role that drives endothelial cell migration upon VEGF induction by forming filopodia, lamellipodia, and stress fibers. VH02 showed inhibitory effect on the phosphorylation of FAK (Y397) with a rapid decay in cytoskeleton integrity. The stimulation of VEGF through VEGFR2 phosphorylation on Tyr1214 leads to SAPK/p28 MAPK activation but it is not a prerequisite of FAK activation [29]. Despite p38 MAPK kinase also associated in cell migration, we did not find significant changes in p38 MAPK kinase signaling. This suggests that p38 MAPK activation was independent of VEGF-stimulated EA.hy926 migration under growth factor-starved condition. Others also reported that FAK activation through VEGFR2 may be independent of the SAPK2/p38 pathway [11,30]. This probably represent that VH02 did not directly affect phosphorylation of p38 MAPK-regulated endothelial cell migration through the VEGF/VEGFR2 complex.

The anti-angiogenic activity of VH02 was confirmed again by in vivo mouse model of vascularization using Matrigel plug assay. A reduction of vascularization in plugs containing VEGF was suppressed by VH02. Immunohistochemical data also revealed that the expression of vascular density and p-Akt intensity were decreased in plugs treated with VH02 or sorafenib (positive drug treatment), which indicated that VH02 significantly diminished angiogenesis in vivo. Besides its angiogenic role, VEGF is also involved in recruitment of pericytes and vascular smooth muscle cells for stabilizing the vessel walls and controlling endothelial cell proliferation [31]. α-SMA is an actin isoform which is the most frequently used as a marker of pericytes. Many studies used these cells to be a new target of antiangiogenenic therapies by identification of α-SMA coverage of vessels. It is also an important factor for detecting the vasculature which was presented in mouse subcutaneous tissue fibroblast [32]. Immunohistochemical examination showed that α-SMA coverage of blood vessels in plugs was more sensitive in VEGF stimulation which was correlated with vascular density and p-Akt intensity. The VEGF stimulates the rate of blood vessel formation which is required for pericyte smooth muscle cells to cover new blood vessels while supporting endothelial stabilization and maturation [31,33]. However, α-SMA was dramatically decreased after the treatment of VH02 or sorafenib which indicates the suppression of pericyte recruitment and angiogenesis. The overall inhibitory effects of VH02 on all the angiogenesis markers studied were influenced by the interaction of factor 1 (the presence of VEGF) and factor 2 (the presence of VH02 or sorafenib).

## 4. Materials and Methods

### 4.1. Reagents and Materials

VH02, 1-((1-(1*H*-indazol-6-yl)-1*H*-1,2,3-triazol-4-yl)methyl)-3-(3-chloromethylphenyl)urea was obtained from Kingkarn Sanphanya, The Department of Pharmaceutical Chemistry, Faculty of Pharmacy, Mahidol University (Bangkok, Thailand). Dulbecco’s modified Eagle medium (DMEM), fetal bovine serum (FBS), penicillin/streptomycin, and trypsin-EDTA were all purchased from Gibco BRL (Gaithersburg, MD, USA). VEGF165aa human recombinant protein, monoclonal antibodies against β-actin, VEGFR2, phospho-VEGFR2 (Tyr1175), Akt, phospho-Akt (Ser473), p38 MAPK, phospho-p38 (Thr180/Tyr182), FAK and phospho-FAK (Tyr397), and 4′-6-diamidino-2phenylindole (DAPI) were provided from Cell Signaling Technology (Beverly, MA, USA). Calcein AM fluorescent dye, 3-(4,5-dimethylthiazol-2-yl)-2,5 diphenyltetrazolium bromide (MTT), anti-mouse, and-rabbit IgG-peroxidase antibodies were ordered from Sigma-Aldrich (St. Louis, MO, USA). Matrigel and CD31 mAb were obtained from BD Bioscience (San Jose, CA, USA). Sorafenib was purchased from Selleckchem (Houston, TX, USA). Alexa Fluor 488-conjugated goat anti-rat, Alexa Fluor 555-conjugated goat anti-rabbit, and Topro-3 iodide were obtained from Invitrogen (Eugene, OR, USA). All other chemicals used in this study were analytical grade.

### 4.2. Cell Culture and Animals

In vitro experiments were first performed in EA.hy926 cells, a human endothelial cell line, purchased from the American Type Culture Collection (ATCC, Rockville, MD, USA). The cells were derived from the fusion of Human umbilical vessel endothelial cells (HUVECs) with the immortal human lung carcinoma cell line A549. EA.hy926 cells were grown in DMEM supplemented with 10% fetal bovine serum (FBS) and 1% penicillin-streptomycin at 37 °C in 5% CO_2_ atmosphere. EA.hy926 cells were allowed to grow about 80% confluent and subcultured about 1 or 2 times per week.

In separate experiments, human umbilical vein endothelial cells (HUVECs) were used to confirm the activity of VH02 in the endothelial cell line EA.hy926. HUVECs were purchased from Lonza (Walkersvile, MD, USA) and cultured in EGM-2 medium supplemented with 2% FBS, hydrocortisone, hFGFB, VEGF, R3-IGF, ascorbic acid, hEGF, GA-1000, and heparin. Cells were used to evaluate the effects of VH02 on VEGFR and Akt phosphorylation using confocal immunohistochemistry. The in vivo study for the antiagiogenic effect of VH02 was performed in six-week-old male C57BL/6 mice. The experiment procedures complied with Regulations for Animal Experiments and Related Activities at Tohoku University (11th). The experiment protocol was approved by the animal ethic committee of Tohoku University, Japan.

### 4.3. Cell Viability Assay

EA.hy926 cells were seeded at a density of 1 × 10^4^ cells/well on 96-well plates (Nunc, ThermoFisher Scientific, Waltham, MA, USA). After overnight culture, cells were treated with or without VEGF (25 ng/mL) and various concentrations (0.01–100 μM) of VH02 for 24 h. Then, MTT solution was added for a final concentration 0.25 mg/mL to each well and incubated at 37 °C for 3 h. At the end of incubation period, MTT solution was removed and the formazan crystal product was solubilized with 100 μL of DMSO with constant shaking for 30 min. The optical density was measured using a Multi-detection microplate reader (Synergy, BioTek, Winooski, VT, USA) at a wavelength of 550 nm. Cell viability was expressed as a percentage relative to control. All experiments were performed in triplicate.

### 4.4. Transwell Migration Assay

The evaluation of endothelial cell migration is the most frequent method for studying chemotaxis in angiogenesis using transwell plates (Costar, #3524, Corning Incorporated, Waltham, NY, USA) with 8 μm diameter pores filters. Briefly, the bottom chambers were filled 600 μL with or without VEGF (25 ng/mL) and various concentrations of VH02. The insert filters were placed into the wells by sterile forceps. Then, cells 1 × 10^4^ cells/well were suspended in 100 μL of media and seeded in the upper chambers. The cultures were allowed to migrate at 37 °C for 16 h. After incubation, the chemoattractant medium in the bottom chambers was removed and replaced with 8 μM Calcein AM fluorescent dye in medium at 37 °C for 45 min. The migrated cells were determined by multi-detection microplate reader at excitation 458 nm and emission at 528 nm (BioTek Instrument, Inc., Winooski, VT, USA).

### 4.5. Cell Adhesion Assay

Endothelial cells were starved in DMEM containing 2% FBS and incubated at 37 °C overnight. The 96-well plates were coated with 50 μL of Matrigel (0.5 μg/mL) and incubated at 37 °C for 1 h. Wells were blocked with 100 μL of 2% BSA in serum-free media for 1 h and washed with PBS 3 times. Next, 2.5 × 10^4^ cells/well containing 0.1% BSA in serum-free media were adhered in wells co-incubated with 25 ng/mL VEGF and different concentrations of VH02 at 37 °C for 2 h. The cultures were washed with PBS to remove non-adherent cells and filled with MTT solution. The adherent cells were measured at 550 nm using a multi-detection microplate reader (version 4.0.1.10, BioTek Instrument, Inc., Winooski, VT, USA). Percentage of adhesion was expressed using the control group as 100%.

### 4.6. Capillary-Like Tube Formation Assay

In vitro capillary-like tube formation on a layer of Matrigel was used to evaluate with differentiation of endothelial cells as part of the angiogenesis process. The 24-well plate was coated 250 μL of Matrigel and incubated at 37 °C for 1 h. EA.hy926 cells (6 × 10^4^ cells/well) were suspended in 1 mL DMEM containing 2% FBS, followed by media with or without VEGF (25 ng/mL) and different concentrations of VH02. After 24 h of incubation, endothelial cells forming capillary-like tubes were photographed by an inverted microscope (Olympus DP20-5E, Olympus Corporation, Tokyo, Japan) with 100× magnification and quantifying the tubular length from at least six randomly selected fields by Cell*B imaging software (version 2.7, Olympus DP20:3.0, Münster, Germany). The percentage of tube length was compared to the control group which represents 100%.

### 4.7. Gelatin Zymography

EA.hy926 cells were starved on 6-well plates, and then DMEM was added to each well containing various concentrations of VH02. VEGF (25 ng/mL) was then added to stimulate cell activity. Cell lysates were collected and the protein samples were separated by 10% SDS-polyacrylamide gels containing 0.1% gelatin. After electrophoresis, the gels were rinsed twice with renaturing buffer (Triton X-100, 2.5% (*v*/*v*) in 0.05 M Tris-HCl pH 8.0, 5 mM Ca_2_Cl) overnight. Then, the gels were incubated twice with developing buffer (0.05 M Tris-HCl pH 8.0, 5 mM Ca_2_Cl) at 37 °C for overnight. Subsequently, the gels were stained with staining solution (0.1% Coomassie Brilliant Blue R-250 in methanol:acetic acid:water, 4.5:1:4.5, *v*/*v*/*v*), and washed with destaining solution. Finally, the bands indicating MMP-2 (72 kDa) were clearly visible in contrast with the blue background and detected by a high resolution scanner (Canon, Canon USA, Inc., Melville, NY, USA). Intensity of bands were quantitated by Image J software (version 1.47, National Institutes of Health (NIH), Bethesda, MD, USA). The MMP-2 activity was expressed as a percentage relative to the control group.

### 4.8. Western Blot Analysis

EA.hy926 cells were starved in medium and treated with 1 and 5 μM VH02 (before exposed to VEGF (50 ng/mL). After treatment, cells were lysed with RIPA buffer supplemented with different kinds of inhibitors (1 mM NaVO4, 10 mM NaF, 1% phosphatase inhibitor cocktail mixed with 1% protease inhibitor cocktail). Proteins samples were separated by 10% SDS-polyacrylamide gel electrophoresis (SDS-PAGE) and transferred to PVDF membranes. The membranes were blocked with 5% BSA in TBS-T (5% *w*/*v* BSA, 1X TBS, 0.1%Tween-20), and probed with primary monoclonal antibody against VEGFR2, p-VEGFR2 (Tyr1175), Akt, p-Akt (Ser473), p38 MAPK, p-p38 MAPK (Thr180/Tyr182), FAK, p-FAK (Tyr397), and β-actin followed by exposure to a horseradish peroxidase-conjugated anti-mouse or anti-rabbit IgG antibody. The membrane was visualized by enhanced chemiluminescence using ECL plus™ western blotting detection reagents and recorded on Gel documentation (GeneGnome5, Integrated Scientific Solutions, San Diego, CA, USA). Band density was quantified by Image J software.

### 4.9. Immunofluorescence Microscopy

EA.hy926 cells (7 × 10^3^ cells/well) were plated on 96 well plates and treated as described above. Cells were fixed with 4% formaldehyde for 15 min, permeabilized with 0.1% Triton X-100 in PBS for 5 min, and blocked with 1% BSA for 30 min at room temperature. Then, cells were incubated with anti–actin antibody (1:200) overnight at 4 °C, and covered with anti–rabbit conjugated Alexa Fluor (1:1000), and counterstained with 4′-6-diamidino-2phenylindole (DAPI). The stained cells were visualized under Olympus immunofluorescence microscope (GE Healthcare, Bucks, UK) with 600× magnification.

To ascertain the effects of VH02 on endothelial cells, the assessment of VEGFR2 and Akt phosphorylation, and F-actin staining were performed in HUVECs. Cell staining was analyzed by high content image analysis using confocal microscopy. HUVECs were seeded on collagen type I coated 8-well culture slides (Costar, #354630, Corning Incorporated) overnight, and serum-starved for 4 h in EGM medium. The cells were treated with VH02 for 30 min before 30 min-VEGF stimulation (20 ng/mL). After fixation, the cells were permeabilized with 0.2% Triton X-100 for 10 min and blocked with 2% FBS in PBS for 40 min. The slides were incubated with antibodies against p-VEGFR2-Tyr1175 and p-Akt (1:300), Alexa Fluor 546-conjugated phalloidin (1:500), and Topro-3 iodide (1:500) at 4 °C overnight. Confocal immunofluorescence microscopy photos were taken with an Olympus FV1000 laser scanning biological microscope at 630× magnification. Photographic images were analyzed for the fluorescent intensity according to p-VEGFR2 and p-Akt staining by MetaMorph software (version 7.7.4, Molecular Devices, Sunnyvale, CA, USA). Actin organization was also shown in the photos.

### 4.10. Matrigel Plug Assay and Immunohistochemistry

Six-week-old male C57BL/6 mice were provided by Prof. Yasufumi Sato in Department of Vascular Biology, Institute of Development, Aging and Cancer (IDAC), Tohoku University, Japan. Mice were subcutaneously injected at opposite iliac regions with 0.6 mL of Matrigel supplemented with 1 μg of VEGF165 with/without 5 μM VH02 or 5 μM sorafenib. After 10 days, the Matrigel plugs were excised and embedded in Tissue Tek OCT (Sakura Finetek Europe B.V., Alphen aan den Rijn, The Netherlands). Cryosections were placed on the glass slides and stained with CD-31, p-Akt, α-SMA mAb, and DAPI. Immunohistochemical staining was performed in Matrigel plugs with Alexa Fluor 488-conjugated goat anti-rat and Alexa Fluor 555-conjugated goat anti-rabbit for 1 h. The vascular expression was photographed by a Leica AF 6500 fluorescence microscope (Wetzlar, Germany) at 250× magnification. Vascular density, p-Akt intensity, and α-SMA coverage on vessel from the histologic sections were quantified in five independent fields of images using the MetaMorph software (version 7.7.4, Molecular Devices, Sunnyvale, CA, USA). The experiment was performed by three mice per groups.

### 4.11. Statistical Analysis

Each assay was performed in triplicate for at least three independent experiments. All data were expressed as the mean ± SEM. Statistical analysis was accomplished by one-way ANOVA followed by Turkey’s test. In addition, the interaction effects between the conditions (PBS or VEGF) and the drugs (vehicle, VH02 or sorafinib) were detected by two-way ANOVA with Bonferroni posttests for multiple comparisons. The statistical significance was considered when *p* value was less than 0.05.

## 5. Conclusions

We demonstrate that VH02 significantly inhibited multifunctional events of VEGF-induced angiogenesis both in vitro and in vivo. VH02 potentially limited VEGFR2-Tyr1175-regulated Akt and FAK signaling pathways that contribute to the regulation both endothelial cell adhesion, migration and tubular formation. Thus, VH02 may represent an appealing novel small molecule VEGFR2 inhibitor for further lead optimization of antiangiogenesis in cancer therapy.

## References

[1] Veikkola T., Karkkainen M., Claesson-Welsh L., Alitalo K. (2000). Regulation of angiogenesis via vascular endothelial growth factor receptors. Cancer Res..

[2] Shibuya M. (2013). Vegfr and type-V RTK activation and signaling. Cold Spring Harbor Perspect. Boil..

[3] Olsson A.K., Dimberg A., Kreuger J., Claesson-Welsh L. (2006). Vegf receptor signaling—In control of vascular function. Nat. Rev. Mol. Cell Biol..

[4] Holmqvist K., Cross M.J., Rolny C., Hagerkvist R., Rahimi N., Matsumoto T., Claesson-Welsh L., Welsh M. (2004). The adaptor protein shb binds to tyrosine 1175 in vascular endothelial growth factor (VEGF) receptor-2 and regulates VEGF-dependent cellular migration. J. Biol. Chem..

[5] Kowanetz M., Ferrara N. (2006). Vascular endothelial growth factor signaling pathways: Therapeutic perspective. Clin. Cancer Res..

[6] Mehta R.R., Yamada T., Taylor B.N., Christov K., King M.L., Majumdar D., Lekmine F., Tiruppathi C., Shilkaitis A., Bratescu L. (2011). A cell penetrating peptide derived from azurin inhibits angiogenesis and tumor growth by inhibiting phosphorylation of VEGFR-2, FAK and Akt. Angiogenesis.

[7] Somanath P.R., Razorenova O.V., Chen J., Byzova T.V. (2006). Akt1 in endothelial cell and angiogenesis. Cell Cycle.

[8] Ikeda Y., Hamano H., Satoh A., Horinouchi Y., Izawa-Ishizawa Y., Kihira Y., Ishizawa K., Aihara K., Tsuchiya K., Tamaki T. (2015). Bilirubin exerts pro-angiogenic property through Akt-eNOS-dependent pathway. Hypertens. Res. Off. J. Jpn. Soc. Hypertens..

[9] Cole C.L., Hansen S.U., Barath M., Rushton G., Gardiner J.M., Avizienyte E., Jayson G.C. (2010). Synthetic heparan sulfate oligosaccharides inhibit endothelial cell functions essential for angiogenesis. PLoS ONE.

[10] Huang S.W., Lien J.C., Kuo S.C., Huang T.F. (2012). Antiangiogenic mechanisms of PJ-8, a novel inhibitor of vascular endothelial growth factor receptor signaling. Carcinogenesis.

[11] Lamalice L., le Boeuf F., Huot J. (2007). Endothelial cell migration during angiogenesis. Circ. Res..

[12] Bellou S., Pentheroudakis G., Murphy C., Fotsis T. (2013). Anti-angiogenesis in cancer therapy: Hercules and hydra. Cancer Lett..

[13] Al-Husein B., Abdalla M., Trepte M., Deremer D.L., Somanath P.R. (2012). Antiangiogenic therapy for cancer: An update. Pharmacotherapy.

[14] Agarwal R., Koenig K., Rohren E., Subbiah V. (2014). Combined antiangiogenic and mammalian target of rapamycin inhibitor targeted therapy in metaplastic breast cancer harboring a PIK3CA mutation. J. Breast Cancer.

[15] Li J., Li S., Chen R., Yu H., Lu X. (2015). The prognostic significance of anti-angiogenesis therapy in ovarian cancer: A meta-analysis. J. Rvarian Res.

[16] Patel R.R., Sengupta S., Kim H.R., Klein-Szanto A.J., Pyle J.R., Zhu F., Li T., Ross E.A., Oseni S., Fargnoli J. (2010). Experimental treatment of oestrogen receptor (ER) positive breast cancer with tamoxifen and brivanib alaninate, a VEGFR-2/FGFR-1 kinase inhibitor: A potential clinical application of angiogenesis inhibitors. Eur. J. Cancer.

[17] Prager G.W., Poettler M., Unseld M., Zielinski C.C. (2012). Angiogenesis in cancer: Anti-VEGF escape mechanisms. Transl. Lung Cancer Res..

[18] Yu H.K., Lee H.J., Yun S.J., Lee S.J., Langley R.R., Yoon Y., Yi L.S., Bae D.S., Kim J.S., Kim S.J. (2014). Antiangiogenic therapy with human apolipoprotein(a) kringle v and paclitaxel in a human ovarian cancer mouse model. Transl. Oncol..

[19] Sanphanya K., Wattanapitayakul S.K., Phowichit S., Fokin V.V., Vajragupta O. (2013). Novel VEGFR-2 kinase inhibitors identified by the back-to-front approach. Bioorg. Med. Chem. Lett..

[20] Eldehna W.M., Abou-Seri S.M., El Kerdawy A.M., Ayyad R.R., Hamdy A.M., Ghabbour H.A., Ali M.M., Abou El Ella D.A. (2016). Increasing the binding affinity of vegfr-2 inhibitors by extending their hydrophobic interaction with the active site: Design, synthesis and biological evaluation of 1-substituted-4-(4-methoxybenzyl)phthalazine derivatives. Eur. J. Med. Chem..

[21] Wu C., Wang M., Tang Q., Luo R., Chen L., Zheng P., Zhu W. (2015). Design, synthesis, activity and docking study of sorafenib analogs bearing sulfonylurea unit. Molecules.

[22] Diez-Cecilia E., Kelly B., Perez C., Zisterer D.M., Nevin D.K., Lloyd D.G., Rozas I. (2014). Guanidinium-based derivatives: Searching for new kinase inhibitors. Eur. J. Med. Chem..

[23] Deryugina E.I., Quigley J.P. (2015). Tumor angiogenesis: MMP-mediated induction of intravasation- and metastasis-sustaining neovasculature. Matrix Biolog..

[24] Pang X., Yi Z., Zhang X., Sung B., Qu W., Lian X., Aggarwal B.B., Liu M. (2009). Acetyl-11-keto-beta-boswellic acid inhibits prostate tumor growth by suppressing vascular endothelial growth factor receptor 2-mediated angiogenesis. Cancer Res..

[25] Hseu Y.C., Chen S.C., Lin W.H., Hung D.Z., Lin M.K., Kuo Y.H., Wang M.T., Cho H.J., Wang L., Yang H.L. (2011). *Toona sinensis* (leaf extracts) inhibit vascular endothelial growth factor (VEGF)-induced angiogenesis in vascular endothelial cells. J. Ethnopharmacol..

[26] Palmieri D., Aliakbarian B., Casazza A.A., Ferrari N., Spinella G., Pane B., Cafueri G., Perego P., Palombo D. (2012). Effects of polyphenol extract from olive pomace on anoxia-induced endothelial dysfunction. Microvasc. Res..

[27] Hu Y.L., Lu S., Szeto K.W., Sun J., Wang Y., Lasheras J.C., Chien S. (2014). Fak and paxillin dynamics at focal adhesions in the protrusions of migrating cells. Sci. Rep..

[28] Chen J.Y., Tang Y.A., Huang S.M., Juan H.F., Wu L.W., Sun Y.C., Wang S.C., Wu K.W., Balraj G., Chang T.T. (2011). A novel sialyltransferase inhibitor suppresses fak/paxillin signaling and cancer angiogenesis and metastasis pathways. Cancer Res..

[29] Lamalice L., Houle F., Huot J. (2006). Phosphorylation of TYR1214 within VEGFR-2 triggers the recruitment of NCK and activation of FYN leading to SAPK2/P38 activation and endothelial cell migration in response to VEGF. J. Biol. Chem..

[30] Rousseau S., Houle F., Kotanides H., Witte L., Waltenberger J., Landry J., Huot J. (2000). Vascular endothelial growth factor (VEGF)-driven actin-based motility is mediated by VEGFR2 and requires concerted activation of stress-activated protein kinase 2 (SAPK2/P38) and geldanamycin-sensitive phosphorylation of focal adhesion kinase. J. Biol. Chem..

[31] Ribatti D., Nico B., Crivellato E. (2011). The role of pericytes in angiogenesis. Int. J. Dev. Biol..

[32] Tonino P., Abreu C. (2011). Microvessel density is associated with VEGF and α-SMA expression in different regions of human gastrointestinal carcinomas. Cancers (Basel).

[33] Lee E., Pandey N.B., Popel A.S. (2014). Lymphatic endothelial cells support tumor growth in breast cancer. Sci. Rep..

